# NON-CELIAC GLUTEN/WHEAT SENSITIVITY (NCG/WS). A BRAZILIAN CASES SERIES

**DOI:** 10.1590/S0004-2803.24612025-104

**Published:** 2026-05-22

**Authors:** Lorete Maria da Silva KOTZE, Luiz Roberto KOTZE, Eloisa Medeiros NISIHARA, Renato NISIHARA

**Affiliations:** 1 Universidade Federal do Paraná, Curso de Medicina, Curitiba, Brasil.; 2 Universidade Positivo, Departamento de Medicina, Curitiba, Paraná, Brasil.; 3 Faculdade Evangélica Mackenzie do Paraná, Curso de Medicina, Curitiba, Paraná, Brasil.

**Keywords:** Non-celiac gluten sensitivity, differential diagnosis, gluten-free diet, gastrointestinal diseases, Sensibilidade ao glúten não celíaca, diagnóstico diferencial, dieta isenta de glúten, doenças gastrointestinais

## Abstract

**Background:**

Non-celiac gluten/wheat sensitivity (NCGWS) is a varied disorder characterized by symptoms related to gluten intake, without celiac disease (CD) or wheat allergy. In Brazil, data on patients with NCGWS remain limited. Objectives: To describe the clinical, serological, and histopathological features of Brazilian patients diagnosed with NCGWS and to compare findings between sexes.

**Methods:**

A retrospective study was conducted using medical records of 36 patients diagnosed with NCGWS between 2015 and 2025 at a gastroenterology clinic in Curitiba, Brazil. Diagnosis was based on excluding CD and wheat allergy, clinical improvement after a gluten-free diet (GFD), and normal or Marsh I duodenal histology. Serology (anti-endomysium/anti-transglutaminase antibodies) and duodenal biopsies were analyzed. Symptoms, comorbidities, and demographic data were compared by sex.

**Results:**

Most patients were women (83.3%). All had negative CD serology. Duodenal biopsies showed Marsh 0 (80% of females, 100% of males) or Marsh I changes. Gastrointestinal symptoms included flatulence, abdominal distension, and reflux; diarrhea was more common in males (*P*=0.0017). All patients reported symptom improvement after two months on a GFD.

**Conclusion:**

In this Brazilian cohort, NCGWS was more frequent in women. Accurate diagnosis relies on histological, serological, and especially thorough clinical evaluation. Multidisciplinary follow-up and personalized dietary guidance are vital for optimal patient outcomes.

## HIGHLIGHTS

• Non-celiac gluten/wheat sensitivity (NCGWS) was more prevalent in women, although clinical presentation did not differ between sexes.

• Histological analysis (Marsh 0 or I) and negative CD serology were key to excluding celiac disease in all patients.

• A gluten-free diet led to symptom improvement in all cases, confirming the clinical diagnosis of NCGWS.

## RESUMO

**Contexto:**

A sensibilidade ao glúten/trigo não celíaca (NCGWS) é uma condição heterogênea caracterizada por sintomas relacionados à ingestão de glúten, na ausência de doença celíaca (DC) ou alergia ao trigo. No Brasil, dados sobre pacientes com NCGWS ainda são escassos.

**Objetivos:**

Avaliar os achados clínicos, sorológicos e histopatológicos de pacientes brasileiros diagnosticados com SGTNC e comparar os achados entre os sexos.

**Métodos:**

Estudo retrospectivo realizado por meio da análise de prontuários de 36 pacientes diagnosticados com SGTNC entre 2015 e 2025 em uma clínica de gastroenterologia em Curitiba, Brasil. O diagnóstico baseou-se na exclusão de DC e alergia ao trigo, melhora clínica após dieta isenta de glúten (DIG) e histologia duodenal normal ou Marsh I. Foram analisadas sorologia (anticorpos antiendomísio/anti-transglutaminase) e biópsias duodenais. Sintomas, comorbidades e dados demográficos foram comparados entre os sexos.

**Resultados:**

A maioria dos pacientes era do sexo feminino (83,3%). Todos apresentaram sorologia negativa para DC. As biópsias duodenais mostraram alterações Marsh 0 (80% das mulheres, 100% dos homens) ou Marsh I. Os sintomas gastrointestinais incluíram flatulência, distensão abdominal e refluxo; a diarreia foi mais comum nos homens (*P*=0,0017). Todos os pacientes relataram melhora dos sintomas após dois meses em DIG.

**Conclusão:**

Nesta coorte brasileira, a NCGWS foi mais frequente em mulheres. A avaliação clínica cuidadosa, aliada aos exames histológicos e sorológicos, é essencial para o diagnóstico correto. O acompanhamento multidisciplinar e a orientação dietética individualizada são cruciais para melhores desfechos clínicos.

## INTRODUCTION

The Oslo definitions for gluten-related diseases classify these disorders based on their pathogenesis into three categories: autoimmune (celiac disease, dermatitis herpetiformis, and gluten ataxia), allergic (wheat allergy), and non-autoimmune, non-allergic (non-celiac gluten sensitivity)^1^. As fructan, rather than gluten, can induce symptoms in patients with self-reported non-gluten sensitivity, Manza et al. suggested naming this syndrome as non-celiac gluten/wheat sensitivity (NCGWS)^2^. To date, diagnostic biomarkers are lacking due to a genetic background predisposing NCGWS^3^. The diagnosis is made after excluding IgE-mediated wheat allergy (WA) and celiac disease (CD). NCGWS remains a heterogeneous and controversial clinical entity with challenges in both recognition and management^3^.

The exact prevalence is unknown, probably due to the lack of biomarkers. It is more commonly reported in women, in first-degree relatives of patients with CD, and more often in adults than in children^3^. In Brazil, there are few papers on this topic^4^.

The purpose of this study is to present a comprehensive profile of clinical and laboratory findings in Brazilian patients diagnosed with NCGWS, comparing the findings between sexes.

## METHODS

### Design and ethical issues

This retrospective study was approved by the local Research Ethics Committee under protocol 6.566.826 and was conducted in accordance with the Good Clinical Practice Guidelines and the Declaration of Helsinki. The study involved a review of patients’ clinical charts and was carried out over a ten-year period (January 2015 to January 2025). All patients were attended by the same physician at a reference Gastroenterology medical office in Curitiba, Paraná, Brazil. Informed consent for participation was obtained from all patients.

### Patients recruitment

Patients on a gluten-containing diet reporting symptoms themselves were included. According to Cárdenas et al.^5^, the definition of NCGWS first relies on excluding CD and wheat allergy, and second on the patient’s responsiveness to a gluten-free diet (GFD).

Thus, in this study, the diagnosis of NCGWS was established based on history, signs and symptoms, physical examination, and serological findings showing negative results for autoantibodies anti-endomysium-IgA and/or anti-transglutaminase antibodies-IgA, following the determination of serum IgA levels. All patients underwent upper gastrointestinal endoscopy, with histopathological findings from duodenal biopsies classified according to the Marsh system^6^.

Patients with a prior diagnosis of IgE WA, those on GFD, or with incomplete records were excluded from the study.

### Data collection

All data were collected at the time of diagnosis. Clinical charts were reviewed, and the included data comprised demographic and anthropometric parameters such as age, age at symptom onset, diagnostic delay, BMI, general, digestive and extra-digestive symptoms, and anemia. Bone disease was evaluated using dual-energy X-ray absorptiometry (DXA) at the lateral distal femur and anterior-posterior spine, along with HLA DQ2 and HLA DQ8 genotyping. Familial gluten-related disorders and other previously diagnosed comorbidities were self-reported.

### Data analysis

Data were collected in frequency tables. Statistical analyses were performed using SPSS version 17.0. The Kolmogorov-Smirnov and Shapiro-Wilk tests were used to evaluate data normality. Continuous variables were expressed as medians and interquartile ranges (IQRs), and compared using the non-parametric Mann-Whitney test. Categorical variables were expressed as percentages and compared with Fisher’s exact test or Chi-squared test, as appropriate. *P* values less than 0.05 were considered statistically significant.

## RESULTS

Thirty-six patients with a mean age of 37.3±10,45 years (range 17 to 69 years) were studied. Table 1 shows the demographic and clinical characteristics of the sample cases. The majority of the cases treated were women (83.3%); however, no differences were observed in the clinical signs and symptoms between sexes.

**TABLE 1:** Demographical and clinical characteristics of sample cases.

	Total n=36
Females	30 (83.3%)
Age (mean±SD) years	37.3±10.45
<20 years	3 (8.3%)
21 to 40 years	17 (42.2%)
41 to 60 years	15(41.6%)
>60 years	1 (2.7%)
Delay diagnosis (median -IQR) years	4 (1-9)
<1 year	5 (15.6%)
1 - 5 years	17 (53.1%)
6- 10 years	5 (15.6%)
> 10 years	5 (15.6%)
Unknown	4 (12.5%)
Body mass index (mean±SD) Kg/m^2^	23.2±3.57
Underweight	1 (2.7%)
Normal weight	27 (75.1%)
Overweight	6 (16.8%)
Obese (degree 1)	2 (5.4%)

All the females showed normal IgA levels, and one man was IgA deficient (serology tested with IgG class). All cases tested negative for antiendomysium and/or anti-transglutaminase antibodies detection. 

Table 2 shows the gastrointestinal complaints/signs referred by the studied patients that can present concomitant several symptoms. Only diarrhea was significantly more frequent in male patients (*P*=0.0017).

**TABLE 2:** Gastrointestinal symptoms/signs in 36 patients with NCGWS.

Symptom	Female (n=30) %	Male (n=6) %	***P* value**
Flatulence	86.7	83.3	0.75
Abdominal distension	80.0	100.0	0.44
Gastroesophagic reflux	53.5	50.0	0.62
Epigastric pain	53.3	16.7	0.19
Abdominal pain	53.3	50.0	0.82
Mal digestion	50.0	50.0	1.0
Nausea	33.3	16.7	0.7
Constipation	33.3	0	0.30
Aphtas	16.3	16.7	0.89
Diarrhea	13.3	66.7	0.0017
Vomit	3.3	0	0.7

HLA-DQ2 and HLA-DQ8 testing was conducted in 34 cases. The absence of both HLA-DQ2 and HLA-DQ8 was found in 57.1% of female cases and 33.3% of male cases. HLA-DQ2 was positive in 32.1% of cases (only females), while HLA-DQ8 was positive in 10.7% of females and 66.7% of males.

Regarding histopathological findings from duodenal biopsies, 24 out of 30 women (80%) exhibited normal architecture with a median of 12.9% intraepithelial lymphocytes (IEL) (range 5 to 22) and were classified as Marsh 0. The remaining 20% were classified as Marsh I with normal architecture, with a median IEL of 32.2% (range 30 to 36%). All the men were classified as Marsh 0, with 9% IEL (range, 1-14%) and normal architecture.

Table 3 displays the main general and additional digestive complaints of the studied patients. Females more commonly exhibited psychiatric symptoms and autoimmune diseases, although the difference was not statistically significant.

**TABLE 3:** Main general and extra digestive complaints of the studied patients.

	Female (n=30) %	Male (n=6) %
**General symptoms**		
Fatigue	13.3	0
Articular pain	13.3	0
Weight loss	10.0	0
Weight gain	10.0	0
**Nutritional imbalance**		
Anemia	16.7	0
Osteopenia femur	26.7	0
Osteopenia spine	20.0	0
**Alimentary**		
Lactose intolerance	40.0	66.7
Cow milk protein allergy	3.3	0
**Neurological**		
Migraine	23.3	16.7
Headache	10.0	0
**Psychiatric manifestations**		
Depression	50.0	33.3
Anxiety	30.0	16.7
**Autoimmune diseases**		
Hashimoto thyroiditis	20.0	16.7
Alopecia areata	6.6	0
Vitiligo	3.3	0
Rheumathoid arthritis	3.3	0

Note: all the comparison between sexes *P*>0.05.

Regarding familial cases of gluten-related disorders, one woman reported a sister with CD, and another woman mentioned a mother and nephew with CD (6.7%). Among males, one man reported a sister with CD (16.7%).

The consumption of beer was self-reported as the cause of abdominal distention in 4 out of 6 (66.7%) males and 6 out of 30 (20%) females. No patients used tobacco.

The patients were treated with a GFD and followed for two months initially, during which all showed clinical improvement.

## DISCUSSION

NCGWS is virtually unknown in Brazil, with no publications on it in the literature. Our study highlights the importance of this research and draws attention to this condition in the differential diagnosis with CD and irritable bowel syndrome (IBS).

NCGWS appears to be more common in women than in men, as our study showed, and also more prevalent among first-degree relatives of CD patients, as reported in the present study. The women were in young adult age.

Given the significant overlap of symptoms with irritable bowel syndrome (IBS), diagnosing NCGWS can be challenging. According to diagnostic criteria consensus^3^, NCGWS was defined as: 1. self-reported gluten intolerance as indicated by the patients in this study; 2. negative CD serology, which was present in all our patients; and 3. absence of villous atrophy in duodenal biopsies, as we demonstrated. Therefore, histological findings were crucial in ruling out CD. Despite the availability of sensitive and specific serological tests, the histopathological features observed in mucosal biopsies remain essential for diagnosis when CD is suspected or needs to be excluded. Robert et al. recently also provided arguments supporting continued reliance on biopsy^7^. The histological changes induced by gluten in the duodenal mucosa of NCGWS patients are poorly understood^8^. The healthy duodenal mucosa is characterized by a villus/crypt ratio greater than 3/1 and fewer than 25 intraepithelial lymphocytes per epithelial cell, corresponding to Marsh^9^. In a previous study in Brazil, we showed that histological patterns correlated with IEL counts^10^. Individuals without digestive disorders exhibited Marsh 0 with an IEL mean of 24.38% (16 to 27%); those with functional diseases showed Marsh 0 or Marsh I with IEL means of 26,48% (18 to 46); and CD patients with Marsh III had IEL means of 43.06% (29 to 64) 10 These findings align with reports by Ferguson and Murray^11^ and support the results of our study.

Most males and 80.0% of females in our study were Marsh 0, indicating normal mucosa as expected in NCGWS. The 20.0% of women classified as Marsh I, with villi architecture within normal limits, a normal villous/crypt ratio of 3:1, and IEL greater than 25/100 epithelial cells, had seronegative antibodies and tested negative for HLA DQ2 / DQ8, so CD was ruled out. Remember that several mimickers (such as drug interactions and medical comorbidities) could cause increased IEL^12^. Our findings are slightly different from Rostami et al.^8^ from Iran, who showed a shorter villus/ratio and higher median IEL in Marsh 0. Nowadays, pathologists are quantifying eosinophils as an additional cause of NCGWS and are describing clustering or linear distributions of IEL^9^ ^,^ ^13^.

HLA haplotypes DQ2/DQ8 can be positive in about 40% of the general population and in 44% of NCGWS according to Molina-Infante et al.^14^. Still, the absence of these genetic markers can exclude CD. In 34 of our patients, the absence of both DQ2 and DQ8 was observed in 57.1% of females and 33,3% of males, aiding in the exclusion of CD. HLA was used to exclude CD, especially considering that some patients have seronegative CD^15^. Therefore, histological findings were essential for excluding CD.

The delay in diagnosis among our patients was primarily 1 to 5 years, similar to the report by Mansueto et al.^16^ in Italian patients. After confirming the diagnosis, nutritional screening to categorize nutritional status will guide treatment on a case-by-case basis^17^. BMI showed a predominance of normal weight in 75.1% of the cases, despite various complaints. However, 16.8% were overweight, probably due to the consumption of processed gluten-free foods high in lipids and sugar, which increases the risk of developing metabolic syndrome^18^.

NCGWS, like CD, can present with a variety of clinical signs including digestive and systemic symptoms^12^ ^,^ ^3^ ^,^ ^19^ ^,^ ^20^. Our findings align with these reports. In Brazil, a survey by Arámburo-Gálvez et al. reported similar results^4^. However, these symptoms are often similar to those seen in IBS, which explains why many patients, including ours, are initially diagnosed with IBS. The main symptoms of NCGWS are similar to those of IBS, but in NCGWS, they typically occur hours to days after wheat consumption. Diarrhea was more frequently reported by men, while women reported more constipation, which differs from Losurdo et al., in Italy^18^. General complaints and anemia listed in TABLE 3 were reported only by women. Osteopenia in the femur/spine among young female patients was notable because these findings were diagnosed before menopause. Additionally, migraine and headache, anxiety, and depression were more commonly reported by female patients^18^ ^,^ ^19^.

NCGWS has an immune-related background. The most common autoimmune disorder (Hashimoto’s thyroiditis) was diagnosed in 20% of females and 16.7% of males, along with other immune-mediated diseases, similar to findings by other authors^18^ ^-^ ^20^. The conclusion is that in patients with NCGWS, a broad approach is relevant because treating concomitant comorbidities can lead to a significantly improved quality of life.

A gluten-free diet (GFD) remains the most effective treatment for NCGWS patients, effectively resolving both digestive and extraintestinal symptoms and improving quality of life (QoL)^3^. A low FODMAP diet could also positively influence treatment management^21^. The recommendations should be based on individual nutritional assessments. Access to a specialized dietitian is essential not only for monitoring adherence to the diet but also for overall nutritional management^17^.

Since patient follow-up is not standardized, we initially evaluated each case after a two-month period on a GFD when patients self-reported symptom improvement, confirming the diagnosis of NCGWS. The question was how long this treatment should be recommended, but regular follow-up will determine this on a case-by-case basis^22^. GFD may not need to be strict or indefinite but should be monitored nutritionally to prevent metabolic complications^17^.

In summary, since NCGWS is a heterogeneous condition diagnosed based on symptoms and without a clear understanding of the disease mechanisms, involving more than one trigger, biomarkers are needed to identify subtypes of NCGWS based on triggers. This would help guide therapies and redefine the condition^23^. The findings reported in the present study support the similarity with cases of NCGWS published in several countries. Additionally, the authors recommend that physicians consider the possibility of many more cases of NCGWS when differentiating this entity from IBS.

## CONCLUSION

In the studied sample of Brazilian patients with NCSWS, there was a higher prevalence of women affected, with no differences in signs and symptoms compared to men. The most common symptoms were flatulence, abdominal distension, and gastroesophageal reflux, and these patients report rapid onset of symptoms after wheat ingestion. Since no specific biomarker for NCGWS has been identified, diagnosis still relies on clinical criteria. A personalized approach, regular follow-up, and support from experienced healthcare professionals are essential for effective patient management.

## Data Availability

Data-available-upon-request
